# Attitude, Practices, and Barriers to Menopausal Hormone Therapy Among Physicians in Saudi Arabia

**DOI:** 10.7759/cureus.52049

**Published:** 2024-01-10

**Authors:** Rayan A Qutob, Abdullah Alaryni, Eysa N Alsolamy, Khalid Al Harbi, Yousef Alammari, Abdulrahman Alanazi, Mohanad Khalid Almaimani, Enad Alsolami, Osamah A Hakami, Asail Ahmed Alammar, Raghad Z Abuthyab, Lana Hesham Alabdulkarim, Razan Khaled Aldeham, Noora Abdulrahman M Alrajhi, Abdulrhman Abdullah AlMufarrej

**Affiliations:** 1 Internal Medicine, College of Medicine, Imam Muhammad Ibn Saud Islamic University, Riyadh, SAU; 2 Medicine, College of Medicine, Imam Muhammad Ibn Saud Islamic University, Riyadh, SAU; 3 Medicine, University of Jeddah, Jeddah, SAU; 4 Internal Medicine, University of Jeddah, Jeddah, SAU; 5 Internal Medicine, College of Medicine, King Abdullah Medical City in Holy Capital (KAMC-HC), Makkah, SAU

**Keywords:** saudi arabia, physicians, hormone therapy, menopause, barriers, practices, attitude

## Abstract

Background: To guarantee the delivery of thorough and scientifically supported menopausal care, it is imperative to allocate resources towards ongoing education and training for physicians. Therefore, it is essential to assess the attitudes, practices, and obstacles faced by physicians in Saudi Arabia when it comes to menopausal hormone replacement therapy (HRT).

Method: An online survey was conducted from June to September 2023 to investigate the attitudes, practices, and barriers of physicians in Saudi Arabia, regarding menopausal HRT. The study population consisted of practicing physicians in Saudi Arabia specializing in gynecology, endocrinology, family medicine, internal medicine, and general practice at various levels, including consultants, senior registrars, and residents. The survey link was distributed to the intended research participants in Saudi Arabia using several social media platforms (Facebook, Twitter, Snapchat, WhatsApp, and Instagram) utilizing a Google Form hyperlink.

Results: A total of 95 physicians participated in this study. A total of 60.0% of the study participants agreed that in general, HRT should be offered to menopausal women who have menopausal symptoms. Besides, around 24.2% of them agreed that in general, HRT should be offered to menopausal women who do not have menopausal symptoms. The most commonly reported methods of obtaining up-to-date information about HRT were Ministry of Health in Saudi Arabia publication and journal articles, contributing 36.8% (n=35) and 24.2% (n=23), respectively. The most commonly reported type of systemic (i.e. non-vaginal) HRT for women with premature menopause (menopause <40 years) without contraindications was combined oral contraceptive pill accounting for 33.7% (n=32). More than half of the study participants (53.6%; n=51) reported experiencing difficulty or barriers related to prescribing HRT. The most commonly reported difficulties and barriers related to HRT prescribing were consumer preferences for complementary/alternative therapies, difficulty explaining HRT risks and benefits to women, and lack of suitable HRT products accounting for 27.4% (n=26), 21.1% (n=20), and 16.8% (n=16), respectively.

Conclusion: The nuanced perspectives of Saudi Arabian physicians regarding HRT for postmenopausal women are revealed in this study. Electronic published societal guidelines and Ministry of Health publications are examples of vital information resources that physicians must have access to. Difficulties associated with the prescription of HRT, including product shortages and consumer preferences, underscore the criticality of confronting obstacles in clinical practice. Additional investigation is suggested in order to enhance physicians' knowledge and implementation of guidelines, specifically for patient cohorts whose medical histories are unique.

## Introduction

Menopause is the biological process in which a woman's monthly menstrual cycle permanently stops for at least 12 consecutive months [1]. According to the World Health Organization (WHO), premenopausal women are individuals who have experienced episodes of irregular bleeding within the last year [1]. Perimenopausal women have experienced either irregular menstruation in the past year or cessation of menstruation for more than three months but less than one year. The onset of menopause exhibits variability among different populations and is subject to the effect of both hereditary and environmental variables. The commonly accepted age range is between 45-55 years, and the occurrence of menopause prior to the age of 40 is referred to as premature menopause [1]. Menopause is a significant stage in a woman's life that is linked to a range of health issues, including as vasomotor, sexual, and psychological symptoms. These symptoms can have a detrimental effect on both the quality of life and productivity [2]. The most common menopausal symptoms among women are hot flashes (87.4%), night sweats (66.6%), and sleeplessness (60.1%) [3]. These symptoms can cause significant distress to many persons. Hormonal replacement therapy (HRT) entails the administration of hormones that are normally depleted during the menopausal phase. Hormone replacement therapy involves the administration of estrogen to alleviate symptoms associated with menopause. If the uterus is still present, progesterone is also added to provide protection for the uterine lining. Routine addition or recommendation of progesterone is not necessary if the uterus is not intact. Administering these medications can effectively diminish or eradicate a wide range of menopausal symptoms, thereby enhancing the overall quality of life [3].

A previous study in Malaysia and other Asian nations, reported misinterpretations or false beliefs of menopause among women [4]. In Asian culture, menopause is commonly regarded as a natural consequence of aging, associated with social status and wisdom within the community. Research conducted on women from Asia and Europe has revealed that younger women generally exhibit more favorable perception in comparison to older women who have undergone menopause [4]. Thai women view menopause as a routine, natural biological process that does not necessitate intervention, but certain Pakistani women regard it as a medical ailment that necessitates medical attention [4].

Healthcare professionals from all fields (except pediatrics) come across patients experiencing menopause. Nevertheless, their understanding and implementation of menopausal hormone therapy (MHT) still varies [5]. A study in Australia assessed the knowledge and attitudes of Australian health professionals, including general practitioners, gynecologists, and endocrinologists, on HRT. The study found that only 52% of the participants had a sufficient level of knowledge about non-hormonal therapy, while 72% had a good understanding of menopausal physiology or HRT. Moreover, there were no significant discrepancies in knowledge detected among various disciplines. However, 91% of health providers expressed their willingness to offer HRT to women who are going through menopausal symptoms. Members of menopause societies demonstrated a higher inclination to endorse HRT for prolonged durations. The main obstacle identified in prescription HRT was the concern expressed by clients regarding the possibility of acquiring breast cancer [5]. According to a survey conducted in 2019 among medical residents specializing in family medicine, internal medicine, and obstetrics and gynecology, only 6.8% of the participants expressed confidence in their ability to effectively handle the medical needs of women going through menopause [6].

A further study conducted in China involving healthcare professionals uncovered a deficiency in their comprehension of the core principles behind HRT, since nearly half of them were incapable of correctly identifying its contraindications. Enhancing clinicians' expertise and education in the management of menopause is essential for providing complete, evidence-based care. Moreover, the study revealed that receiving training on menopause care has a substantial impact on physicians' attitudes, behaviors, and confidence in identifying menopausal symptoms and offering suitable suggestions for symptom management [7].

While many physicians acknowledge the necessity of education on menopausal therapy, there exist notable gaps in knowledge that require attention. Ensuring the provision of comprehensive, evidence-based menopausal care necessitates investing in continuous education and training for physicians. Hence, it is crucial to evaluate attitudes, practices, and barriers of physicians in Saudi Arabia, regarding menopausal HRT.

## Materials and methods

Study design

An online survey was conducted from June to September 2023 to investigate the attitudes, practices, and barriers of physicians in Saudi Arabia, regarding menopausal HRT.

Study population

The study population consisted of practicing physicians in Saudi Arabia specializing in gynecology, endocrinology, family medicine, internal medicine, and general practice at various levels, including consultants, senior registrars, and residents. There were no restrictions regarding the gender or demographic features of the study participants. Physicians of various specialties or those who are not currently practicing medicine in Saudi Arabia were removed.

Sampling technique

This study employed a convenient sampling approach. The survey link was distributed to the intended research participants in Saudi Arabia using several social media platforms (Facebook, Twitter, Snapchat, WhatsApp, and Instagram) utilizing a Google Form hyperlink. In addition, a cover letter was enclosed alongside a consent form. Participation was voluntary, granting individuals the freedom to withdraw as they saw fit. The comments were given anonymously, with no tracking of email addresses or any identifiable information.

Questionnaire tool

This study utilized a pre-existing questionnaire that was created by Yeganeh et al. [5]. The study questionnaire consisted of five components. The initial section was designed to collect data on the physician's demographic features, including: age category, gender, nationality, specialty, level of expertise, years of experience, practice region, practice location, and the weekly number of perimenopausal or postmenopausal female patients they attend to. The second section examined the physicians' attitude toward hormone replacement therapy. The third section analyzed the practices of physicians regarding HRT. This included their ability to stay updated with current HRT recommendations, their preferred method of obtaining up-to-date information about HRT, their preferred duration of systemic HRT for women with premature menopause, their preferred duration of combined HRT for symptomatic women over 50 years old, their preferred duration of estrogen-only HRT for symptomatic women without a uterus over 50 years old, their preferred treatment for hot flashes, their preferred type of systemic HRT for women with premature menopause without contraindications, and their preferred type of systemic HRT for women over 50 years old without contraindications. The fourth section examined the likelihood of administering systemic hormone replacement HRT to a woman experiencing menopause, while the fifth section explored the challenges associated with HRT.

Questionnaire piloting

In the pilot phase, the questionnaire was sent randomly to different locations in Saudi Arabia among a sample of 30 participants to evaluate the face validity of the study tool. The degree to which the items measure or assess what it is intended to measure. These participants did not make any contributions to the findings of the primary investigation.

Ethical approval

The ethical approval for this study was obtained from the ethical research committee of the Institutional Review Board (IRB) of Imam Muhammed Ibn Saud Islamic University (IMSIU), Riyadh, Saudi Arabia, wherein they reviewed and approved this project (HAPO-01-R-0011; Project No.499/2023). Informed consent was obtained from all participants.

Data analysis

The data were analyzed utilizing the Statistical Package for Social Science Software (SPSS), especially version 28 (IBM Corp., Armonk, NY, USA). Numerical values and percentages (%) were used to represent categorical variables.

## Results

Participants’ demographic characteristics

A total of 95 physicians were involved in this study. Around 41.1% (n=39) of them were aged 25-34 years. More than half of them (53.7; n=51) were males. The vast majority of the study participants (82.1%; n=78) were Saudis. Around one-third of the study participants (35.8%; n=34) were gynaecologists. Almost 47.4% (n=45) of the study participants were residents. Around 34.7% (n=33) of the study participants reported that they have an experience of less than five years. More than half of the study participants (65.3%; n=62) reported that they practice in the Central region. Around 30.5% (n=29) of the study participants reported that they work at military, security, or national guard hospitals. More than half of the study participants (55.8%; n=53) reported that the number of perimenopausal or postmenopausal women that they see as patients each week is less than five patients. For further details on the demographic and practice characteristics of the study participants refer to Table 1.

**Table 1:** Participants’ demographic and practice characteristics

Variable	Frequency	Percentage
Age group		
25-34 years	39	41.1%
35-44 years	19	20.0%
45-54 years	16	16.8%
55-64 years	16	16.8%
65 years and older	5	5.3%
Gender		
Males	51	53.7%
Females	44	46.3%
Nationality		
Non-Saudi	17	17.9%
Saudi	78	82.1%
Specialty		
Gynaecologist	34	35.8%
Endocrinologist	14	14.7%
Family medicine	17	17.9%
Internal medicine	16	16.8%
General practitioner	14	14.7%
Level of experience		
Consultant	30	31.6%
Fellow	4	4.2%
Senior registrar	6	6.3%
Registrar	10	10.5%
Resident	45	47.4%
Years of experience		
Less than 5 years	33	34.7%
5-10 years	30	31.6%
11-15 years	18	18.9%
16-20 years	6	6.3%
More than 20 years	8	8.4%
Region of Practice		
Central region	62	65.3%
Eastern region	11	11.6%
Western region	14	14.7%
Northern region	3	3.2%
Southern region	5	5.3%
Place of practice		
Military, security, or national guard hospital	29	30.5%
University hospital	23	24.2%
MOH secondary hospital	14	14.7%
Primary healthcare centre	13	13.7%
Private hospital	10	10.5%
Medical city/specialised hospital	6	6.3%
The number of perimenopausal or postmenopausal women that I see as patients each week		
Less than five patients	53	55.8%
6-10 patients	30	31.6%
11-15 patients	8	8.4%
16-20 patients	2	2.1%
More than 20 patients	2	2.1%

Attitude towards hormone replacement therapy

Table 2 below presents physicians’ attitudes towards hormone replacement therapy. A total of 60.0% of the study participants agreed that in general (agreed or strongly agreed), HRT should be offered to menopausal women who have menopausal symptoms. Besides, around 24.2% of them agreed that in general, HRT should be offered to menopausal women who do not have menopausal symptoms.

**Table 2:** Attitude towards hormone replacement therapy HRT: Hormone replacement therapy

	Strongly agree	Agree	Neutral	Disagree	Strongly disagree
In general, HRT should be offered to menopausal women who have menopausal symptoms.	21.1%	38.9%	37.9%	2.1%	0.0%
In general, HRT should be offered to menopausal women who do not have menopausal symptoms.	4.2%	20.0%	28.4%	40.0%	7.4%

Hormone replacement therapy practices profile

Table 3 below presents hormone replacement therapy practices profile of the study participants. A total of 48.4% (n=46) of the study participants agreed that they find it easy to keep up with current HRT recommendations/guidelines/evidence. The most commonly preferred methods of obtaining up-to-date information about HRT were Ministry of Health publications and journal articles, contributing for 36.8% (n=35) and 24.2% (n=23), respectively. The most commonly preferred duration of systemic (i.e. non-vaginal) HRT for women with premature menopause was one to five years, accounting for 60.0% (n=57). The most commonly preferred duration of systemic (non-vaginal) combined HRT (oestrogen + progesterone) for symptomatic women aged over 50 years was one to five years, accounting for 55.8% (n=53). The most commonly preferred duration of systemic (non-vaginal) oestrogen-only HRT for symptomatic women without a uterus aged over 50 years was one to five years, accounting for 49.5% (n=47). The most commonly preferred treatment for hot flashes was lifestyle modifications (32.6%; n=31).

**Table 3:** Hormone replacement therapy practices profile HRT: Hormone replacement therapy

Variable	Frequency	Percentage
I find it easy to keep up with current HRT recommendations/guidelines/evidence:		
Strongly agree	19	20.0%
Agree	27	28.4%
Neutral	28	29.4%
Disagree	20	21.1%
Strongly disagree	1	1.1%
Preferred method of obtaining up to date information about HRT:		
Ministry of Health publication	35	36.8%
Journal articles	23	24.2%
Saudi Health Council	11	11.6%
Menopause Society website	6	6.3%
Pharmaceutical company medical representatives	6	6.3%
Conferences	4	4.2%
Medical magazine	4	4.2%
Email/e-news	4	4.2%
Webcast	2	2.2%
Preferred duration of systemic (i.e. non-vaginal) HRT for women with premature menopause:		
1-5 years	57	60.0%
6-10 years	8	8.4%
11-15 years	5	5.3%
Until age 50-51 years	9	9.5%
Indefinite	16	16.8%
Preferred duration of systemic (non-vaginal) combined HRT (oestrogen+progesterone) for symptomatic women aged over 50 years:		
1-5 years	53	55.8%
6-10 years	16	16.8%
11-15 years	5	5.3%
Until age 50-51 years	0	0.0%
Indefinite	21	22.1%
Preferred duration of systemic (non-vaginal) oestrogen-only HRT for symptomatic women without a uterus aged over 50 years:		
1-5 years	47	49.5%
6-10 years	5	5.3%
11-15 years	7	7.4%
Until age 50-51 years	5	5.3%
Indefinite	31	32.6%
Preferred treatment for hot flashes:		
Lifestyle modification	31	32.6%
Hormone replacement therapy	28	29.5%
Complementary and alternative therapy (eg herbal, acupuncture)	18	18.9%
Non-hormonal medication (eg venlafaxine, gabapentin)	12	12.6%
Others	6	6.3%
Preferred type of systemic (i.e. non-vaginal) HRT for women with premature menopause (menopause < 40 years) without contraindications:		
Combined oral contraceptive pill	32	33.7%
Oral hormone replacement therapy	30	31.6%
Transdermal patch or gel	4	4.2%
Hormone implant	1	1.1%
Compounded bio-identical hormone therapy	1	1.1%
I prefer not to use hormone replacement therapy	27	28.4%
Preferred type of systemic (i.e. non-vaginal) HRT for women older than 50 years without contraindications:		
Oral hormone replacement therapy	47	49.5%
Compounded bio-identical hormone therapy	7	7.4%
Transdermal patch or gel	6	6.3%
Hormone implant	3	3.2%
I prefer not to use hormone replacement therapy	32	33.7%

The most commonly preferred type of systemic (i.e. non-vaginal) HRT for women with premature menopause (menopause < 40 years) without contraindications was combined oral contraceptive pill accounting for 33.7% (n=32). The most commonly preferred type of systemic (i.e. non-vaginal) HRT for women older than 50 years without contraindications was oral hormone replacement therapy, accounting for 49.5% (n=47).

Likelihood of prescribing systemic HRT to menopausal women

The most commonly agreed upon condition for which physicians would prescribe systemic HRT to a menopausal woman were those who have family history of breast cancer, personal history of breast cancer, and patient has concern about breast cancer, accounting for 48.5% (n=46), 43.2% (n=41), and 40.0% (n=38), respectively, Table 4.

**Table 4:** Likelihood of prescribing systemic HRT to a menopausal woman who has menopausal symptoms stratified by condition HRT: Hormone replacement therapy

	Very likely	Likely	Neutral	Unlikely	Very unlikely
Personal history of breast cancer	37.9%	5.3%	12.6%	14.7%	29.5%
Family history of breast cancer	5.3%	43.2%	20.0%	14.7%	16.8%
Personal history of venous thrombosis	2.1%	9.5%	46.3%	22.1%	20.0%
History of cerebrovascular disease	2.1%	27.4%	28.4%	29.5%	12.6%
History of ischaemic heart disease	4.2%	13.7%	45.3%	20.0%	16.8%
Hyperlipidaemia	4.2%	17.9%	40.0%	24.2%	13.7%
Hypertension	5.3%	25.3%	41.1%	21.1%	7.4%
History of uterine cancer	8.4%	20.0%	38.9%	25.3%	7.4%
Diabetes mellitus	6.3%	18.9%	41.1%	13.7%	20.0%
Obesity	8.3%	29.5%	41.1%	17.9%	3.2%
Patient has concern about breast cancer	11.6%	28.4%	38.9%	15.8%	5.3%

Hormone replacement therapy challenges

Table 5 below presents hormone replacement therapy challenges. More than half of the study participants (53.6%; n=51) agreed that they experience difficulty or barriers related to prescribing HRT. The most commonly reported difficulties and barriers related to HRT prescribing were consumer preferences for complementary/alternative therapies, difficulty explaining HRT risks and benefits to women, and lack of suitable HRT products accounting for 27.4% (n=26), 21.1% (n=20), and 16.8% (n=16), respectively.

**Table 5:** Hormone replacement therapy challenges HRT: Hormone replacement therapy

Variable	Frequency	Percentage	
I experience difficulty or barriers related to prescribing HRT			
Strongly agree	18	18.9%	
Agree	33	34.7%	
Neutral	29	30.5%	
Disagree	9	9.5%	
Strongly disagree	6	6.3%	
The difficulties and barriers related to HRT prescribing:			
Consumer preferences for complementary/alternative therapies	26	27.4%	
Difficulty explaining HRT risks and benefits to women	20	21.1%	
Lack of suitable HRT products	16	16.8%	
Lack of confidence in prescribing HRT	15	15.8%	
Consumer concern regarding use of HRT and breast cancer.	13	13.7%	
Consumer concerns regarding use of HRT and other (non-breast cancer) potential risks	12	12.6%	
Difficulty accessing HRT information for consumers	12	12.6%	
Time constraints when consulting to discuss HRT.	11	11.6%	
Medico-legal consequences of prescribing HRT	8	8.4%	

## Discussion

Hormone replacement therapy is frequently used to alleviate a range of menopausal symptoms [8]. However, HRT can have several negative effects that vary depending on the method of administration and whether it has local or systemic effects [9]. The use of HRT has been a subject of discussion among physicians, and HRT has a complex pattern of risks and benefits [10]. Consequently, this study aims to investigate the attitudes, practices, and obstacles related to menopausal hormone therapy among physicians in Saudi Arabia.

It was suggested that, following a comprehensive assessment by clinicians, HRT is an effective treatment for vasomotor symptoms and the genitourinary syndrome and has been shown to prevent bone loss and fracture [11]. HRT offers potential benefits to menopausal women, including safeguarding against cardiovascular issues, preventing osteoporosis, and improving their overall quality of life. Nevertheless, it is crucial to recognize the apprehensions regarding potential risks associated with HRT, such as an increased likelihood of breast cancer and cardiovascular disease [11].

In fact, physicians have a variety of resources at their disposal for staying current with recommendations and evidence related to HRT [11,12], while in our study, the predominant methods for acquiring current information about HRT were Ministry of Health publications, favored by 36.8%, and journal articles, chosen by 24.2% of the participants. Other information resources may play a crucial role in staying current with recommendations and evidence related to HRT, including the valuable tools provided by the internet, including the Cochrane Library, Medline, and search engines like SUMSearch and TRIP, offering access to systematic reviews and primary research [13]. Moreover, digital guidelines are another important resource, capable of continuous updates and widespread dissemination [14], as well as web-based resources, such as web alerts, medical newsletters, electronic databases, podcasts, and mobile apps, also aid physicians in staying informed about the latest scientific findings [15]. As a result, HRT treatment choice should be based on up-to-date information and targeted to individual women's needs [11,12], where the latest available evidence including the British Menopause Society and the North American Menopause Society can be used to provide guidance to prescribers of HRT and alternatives [11,12].

The duration of hormone replacement therapy for menopausal women varies depending on certain factors, in our study, a total duration of one to five years of HRT was the most commonly preferred duration for systemic (i.e. non-vaginal) HRT for women with premature menopause, for systemic (non-vaginal) combined HRT (oestrogen + progesterone) for symptomatic women aged over 50 years, and for systemic (non-vaginal) oestrogen only HRT for symptomatic women without a uterus aged over 50 years. In fact, most users of hormone replacement therapy have been taking HRT for more than five years [16-19], where systemic hormone therapy initiated early in menopause appeared to have benefits for women under 60 or within 10 years of menopause [20]. Therefore, it is crucial to assess the benefits of systemic HRT in light of individual risk profiles and personal preferences, where treatment duration should be personalized, taking into consideration these factors to ensure optimal outcomes for each individual [21]. Additionally, it is proven that HRT is an effective treatment for hot flashes, yet its utilization is constrained by lack of understanding of therapy risks and benefits [22]. However, the preferred approach selected by 32.6% of the participants, for addressing hot flushes in our study was overwhelmingly centred around lifestyle modifications, where lifestyle modifications, encompassing strategies such as obesity management, exercise, smoking cessation, relaxation techniques, and acupuncture, have demonstrated promise in effectively managing hot flashes [23].

Our study results found that the most commonly preferred type of systemic (i.e. non-vaginal) HRT for women with premature menopause (menopause < 40 years) without contraindications was combined oral contraceptive pill accounting for 33.7%, where the use of low-dose combined oral contraceptive pills (COC) in perimenopausal women can yield multiple positive effects, encompassing pregnancy prevention, regulation of the menstrual cycle, and alleviation of menopausal symptoms [24]. Despite concerns regarding the safety of COC in this age group, existing evidence does not indicate an elevated risk of cardiovascular events or breast cancer [25]. In addition, the most commonly preferred type of systemic (i.e. non-vaginal) HRT for women older than 50 years without contraindications was oral hormone replacement therapy, accounting for 49.5%; indeed, the most favorable outcomes from HRT are observed in symptomatic women aged 50-59 years within 10 years of menopause, and there is a suggestion that low-dose or ultra-low-dose HRT might offer a better safety profile in older women [26]. Also, oral HRT has been linked to cardiovascular benefits, including a decrease in the risk of coronary artery disease [11].

The conditions for which physicians would most commonly prescribe systemic HRT to menopausal women were those with a family history of breast cancer (48.5%), a personal history of breast cancer (43.2%), and patients expressing concerns about breast cancer (40.0%), in fact such practices of prescribing systemic HRT for these groups are a place of a debate, where a study on Korean women found no significant association between HRT history and breast cancer risk [27], as well as the use of HRT in women with a family history of breast cancer is not linked to a significantly increased incidence of breast cancer. However, it is associated with a significantly reduced total mortality rate [28]. On the other hand, physicians often hesitate to prescribe HRT for women with a history of previous breast cancer due to concerns about potentially triggering a recurrence of the disease [11,29].

In our study, a majority of the participants (53.6%) acknowledged facing challenges or encountering barriers in relation to prescribing HRT, where the most frequently cited difficulties and barriers associated with HRT prescribing included consumer preferences for complementary/alternative therapies (27.4%), challenges in explaining the risks and benefits of HRT to women (21.1%), and a shortage of suitable HRT products (16.8%). In fact, research indicates that a significant number of women use and are satisfied with both conventional HRT and complementary therapies for menopausal symptoms [30], where a majority of menopausal women tend to consider or try complementary and alternative medicine as alternatives to HRT [31], and these complementary therapies, such as phytoestrogens, are increasingly popular due to their perceived naturalness [32]. However, the safety and efficacy of these therapies, including their potential interactions with HRT, remain areas of concern [33]. Indeed, the task of explaining the risks and benefits of HRT to women is challenging, since the decision to use HRT requires an individualized and thoughtful approach, influenced by the dual nature of HRT-proven efficacy but accompanied by potential risks, notably an increased risk of breast cancer [11,12]. Therefore, balancing the potential benefits against the associated risks becomes a critical aspect in this decision-making process. Nevertheless, the shortage of suitable HRT products is a global concern [34,35], where this persistent issue has prompted the UK government to impose restrictions on HRT exports as a measure to address the shortage [36,37].

Based on our study, it is recommended that physicians approach HRT for menopausal women with a personalized and carefully considered strategy, weighing the proven benefits against potential risks, including an increased risk of breast cancer and cardiovascular disease. This research identified multiple education opportunities for physicians concerning HRT. These include enhancing physicians' knowledge of HRT duration of therapy and dosage forms. To enhance knowledge, clinicians should utilize a diverse range of resources, including Ministry of Health publications, journal articles, electronic guidelines, the North American Menopause Society, and internet-based tools. Addressing challenges in HRT prescribing, such as consumer preferences for complementary therapies and a shortage of suitable products, is crucial. Effective communication on risks and benefits is vital, considering individual preferences. Regarding treatment duration, personalization based on individual risk profiles is recommended. Healthcare professionals need better menopause management education. Physicians need further education on how to implement the most recent guidelines' recommendations [11]. Further research is needed to guide the prescription of HRT for women with a history of breast cancer. Overall, maintaining a balanced and informed approach is essential in navigating the complexities of HRT decision-making.

Study limitations

The main limitation of this study is the online cross-sectional study design, which might have missed some of our targeted population who don't have access to online platforms (older population); which might have affected the generalizability of our findings. Besides, the cross-sectional design restricted our ability to examine causality among the study variables. In this study, we did not estimate the number of participants who received the study invitation; therefore, we are unable to estimate the response rate. Sampling bias is also a potential limitation of this study as this study employed a convenient sampling approach, which may introduce bias into the results. The small sample size is another limitation. A larger sample size would provide more robust and generalizable results. The data collected from the online survey were self-reported by the participating physicians, which may be subject to recall bias or misreporting. While the questionnaire used in the study was pre-existing, its validity and reliability in the context of Saudi Arabian physicians may not have been fully established. Therefore, our study findings should be interpreted carefully.

## Conclusions

This study reveals the nuanced perspectives of Saudi Arabian physicians on HRT for menopausal women. Physicians reported practices and preferences related to HRT were different from previous studies. International and national information resources, including Ministry of Health in Saudi Arabia publications and electronic societal guidelines, are crucial for keeping physicians informed. Challenges in HRT prescribing, such as consumer preferences and product shortages, highlight the importance of addressing barriers in clinical practice. Physicians at all levels need better knowledge about HRT safety, indications, and contraindications. Physicians should be educated more on HRT therapy and its benefits for women with menopausal symptoms. Besides, physicians should be educated more on the preferred duration and dosage form for HRT therapy.
